# Dendritic Cell Development: A Choose-Your-Own-Adventure Story

**DOI:** 10.1155/2013/949513

**Published:** 2013-02-18

**Authors:** Amanda J. Moore, Michele K. Anderson

**Affiliations:** ^1^Division of Biological Sciences, Sunnybrook Research Institute, 2075 Bayview Avenue, Toronto, ON, Canada M4N 3M5; ^2^Department of Immunology, University of Toronto, Toronto, ON, Canada M5S 1A8

## Abstract

Dendritic cells (DCs) are essential components of the immune system and contribute to immune responses by activating or tolerizing T cells. DCs comprise a heterogeneous mixture of subsets that are located throughout the body and possess distinct and specialized functions. Although numerous defined precursors from the bone marrow and spleen have been identified, emerging data in the field suggests many alternative routes of DC differentiation from precursors with multilineage potential. Here, we discuss how the combinatorial expression of transcription factors can promote one DC lineage over another as well as the integration of cytokine signaling in this process.

## 1. Introduction

Dendritic cells (DCs) are professional antigen-presenting cells that bridge the gap between the innate and adaptive immune systems by acting as sentinels throughout the body to capture, process, and present antigen to T cells. Their ability to distinguish between self and nonself molecules allows them to deliver tolerizing or activating signals to T cells accordingly. Scientific exploration of DCs has become increasingly complex with the recognition that DCs exist as a heterogenous mixture of populations. Named for their cellular size and morphology [1], DCs all share the ability to activate naïve T cells but exhibit unique functions within each subset. These DC populations have primarily been defined by their combinatorial cell surface marker expression, but they also differ in their developmental origins, transcriptional regulation, patterns of migration or residence, and anatomical and microenvironmental localization. DCs can be broadly classified as two major subsets: the inflammatory or infection-derived DCs, which develop from monocytes in response to stimulation, and the steady-state DCs, which are present at all times. The DCs present under steady state conditions include CD8^+^ and CD8^−^ conventional DCs (cDCs), plasmacytoid DCs (pDCs), and migratory CD103^+^ CD11b^−^ DCs, CD103^−^ CD11b^+^ DCs, and Langerhans cells (LCs) (Table 1). The CD8^−^ cDCs can be further classified as CD4^+^ or CD4^−^ DCs, which both express high levels of CD11b [2]. However, the majority of gene perturbation analyses that have examined CD8^+^ cDCs, CD8^−^ cDC, and pDCs as well as global gene analysis have shown mostly congruent gene expression between the CD4^+^ and CD4^−^ subsets [3]; thus, we will classify CD4^+^ and CD4^−^ DCs as CD8^−^ DCs for simplicity.

 The cDCs and pDCs are found throughout the primary and secondary lymphoid organs. In the spleen and lymph nodes (LNs), the CD8^−^ cDCs constitute the majority of the resident DCs, whereas the CD8^+^ cDCs are the predominant DC subset within the thymus. Initially termed interferon-producing cells (IPCs) in humans, pDCs are known for their hallmark function of detecting virus by TLR7 or TLR9 and producing vast amounts of type I interferons [4, 5]. CD8^+^ cDCs are specialized for efficient cross-presentation of antigen to CD8^+^ T cells, resulting in heightened viral and antitumor responses [6, 7]. Since cross-presentation has been associated with more efficient negative selection, it is likely that the higher proportion of CD8^+^ cDCs within the thymus can be attributed to this unique function [8, 9]. Although thymic DCs (tDCs) can participate in negative selection [10], a definitive requirement for tDCs in this process is still debated [11]. CD8^−^ cDCs are distinguished by their superior phagocytic abilities which lead to enhanced presentation of antigen to MHC class II-restricted CD4^+^ T cells [12, 13].

In nonlymphoid organs, the roles of CD103^+^ CD11b^−^ DCs and CD103^−^ CD11b^+^ DCs mirror the specialized functions of CD8^+^ and CD8^−^ cDCs, respectively. A unique CD103^+^ CD11b^+^ subset also exists, but only in the lamina propria of the intestine [14]. There are also CD103^+^ (dermal DCs) and CD11b^+^ subsets, which monitor peripheral locations and migrate to draining LNs upon activation. The epithelium-resident LCs are another type of DC that responds to activation by migrating to skin-draining LNs where they present antigen to T cells [15, 16]. 

Human DC subsets within the peripheral blood, where pDCs were first discovered, have been extensively studied, but due to practical limitations lymphoid and nonlymphoid tissue-resident DCs are less well understood. However, the vast amounts of data on murine DC subsets have enabled the identification of equivalent human DC populations by correlative functional characterization, gene profiling, and by the identification of genetic mutations resulting in human DC deficiency (reviewed in [17]) [18–22]. A summary of the designations of murine DC subsets as defined by cell surface molecules and the transcriptional regulators involved in the development of each subset is shown in Table 1. The equivalent human populations of cDCs and pDCs are also summarized.

Although DC classification has historically been defined by cell surface markers, it is important to note that molecules, such as B220, CD8*α*, and DEC-205, can be upregulated or downregulated following activation or stimulus. DC researchers remain in a quandary, as it is difficult to ascertain whether the identification of DC subsets by surface marker expression relates to discrete lineages or specific physiological states due to the plasticity of DC populations. For example, cells displaying a pDC phenotype can upregulate CD8*α*, downregulate B220, and manifest a classical DC morphology upon stimulation with CpG [23, 24]. Similarly, although Langerin is historically a marker for skin-resident or migratory DCs, it was recently shown that the majority of CD8^+^ tDCs also express Langerin [25]. In order to truly understand the capabilities of these DC subsets, we will need to move beyond cell surface markers and define the transcriptional regulators that govern their genetic programming. Here, we will focus on the origins and development of CD8^+^ cDCs, CD8^−^ cDCs, and pDCs, with an emphasis on the transcription factors that control lineage choice and differentiation of these DC subsets.

## 2. Dendritic Cell Progenitors

### 2.1. Laying the Groundwork

Although considerable advances have been made in identifying upstream DC precursors in the past decade, much is still unknown. An understanding of the cellular origins of peripheral lymphoid tissue-resident DCs largely began with the advent of the identification of common lymphoid progenitors (CLPs; Lin^−^ IL-7R^+^ Thy-1^−^ Sca-1^int^ c-Kit^int^) and common myeloid progenitors (CMPs; Lin^−^ IL-7R*α* ^−^ Sca-1^−^ c-Kit^+^ FcR*γ*RII/III^lo^ CD34^+^) at the turn of the century [36, 37]. Following intravenous injections into lethally irradiated recipients, CLPs, CMPs, and granulocyte/macrophage precursors (GMPs; Sca-1^−^ c-Kit^+^ IL-7Ra^−^ FcR*γ*RII/III^+^ CD34^+^) all gave rise to splenic DCs [38–40]. Interestingly, CLPs produced greater absolute numbers of DCs and a higher proportion of CD8^+^ DCs in the spleen than CMPs [40]. Moreover, Flt3, a cytokine receptor required for peripheral lymphoid tissue DC development [41], was expressed at higher levels on CLPs relative to CMPs [42]. Fate-mapping mice, in which cells expressing IL-7R were irreversibly labeled with YFP, revealed that only one tenth of thymic and splenic CD8^+^ and CD8^−^ cDCs had arisen from IL-7R^+^ precursors, suggesting that most of these cells did not arise from CLPs [43]. In contrast, the majority of thymic and splenic pDCs were YFP^+^. However, these pDCs also expressed *IL7r* mRNA, thereby confounding the determination of whether they had arisen from CLPs. Nevertheless, the reconstitution of irradiated recipients with each of these precursors did not collectively regenerate the same numbers of DCs observed following injection of whole bone marrow, foreshadowing the presence of unidentified DC precursor(s) [40].

### 2.2. The Common DC Precursor with Conventional and Plasmacytoid DC Potential

The identification of a more defined DC precursor was inspired by observations that Flt3 ligand (Flt3L), GM-CSF, and M-CSF could support DC development *in vitro*. Subsequent pursuits of DC lineage precursors identified a bipotent macrophage/DC precursor (MDP; Lin^−^ c-Kit^hi^ CD115^+^ CX_3_CR1^+^ Flt3^+^) [44] that gives rise to a common DC precursor (CDP; Lin^−^ c-Kit^lo^ CD115^+^ CX_3_CR1^+^ Flt3^+^) [45–47] in which macrophage lineage potential is lost. The CDP can then diverge into pre-cDCs (Lin^−^ CD11c^+^ MHC class II^−^ SIRP*α* ^int^ Flt3^+^) or a yet unidentified precursor leading to pDCs [47]. All cDC populations in lymphoid organs and tissue-resident CD103^+^ DCs can arise from pre-cDCs [47, 48]. However, this pathway is not mutually exclusive from the CLP or CMP pathways nor does it eliminate other alternative pathways of DC differentiation. Instead, it appears that there are different developmental routes that converge to give rise to the same functional subsets of DCs.

### 2.3. Development of Thymic Dendritic Cells

There has been much controversy over the origins of the three major subsets of tDCs (CD8^+^ cDCs, CD8^−^ cDCs, and pDCs) and whether they develop within the thymus [25, 35, 43, 49–51]. There are three major developmental routes by which these tDCs could arise. First, they could develop extrathymically and migrate in as mature DCs. Secondly, they could arrive in the thymus as committed DC precursors and differentiate within the thymus. Thirdly, they could arise within the thymus from an uncommitted precursor that shares T cell and DC potential. Development into tDCs has been proposed to occur outside of the thymus for some subsets, namely, CD8^−^ cDCs, and pDCs [52, 53]. In fact, bone marrow-derived MDP, CDP, and pre-DC populations can give rise to tDCs following intravenous injections [25]. In addition, a model of CCR9-dependent pDC migration to the thymus suggests that peripheral self-antigen can be transported from the periphery to the thymus by pDCs and cDCs, in the absence of activation [54]. However, other studies have suggested that intrathymic DC development occurs, as well [25, 35]. The environment of the thymus, which is the primary site of T cell development, provides a vastly different set of microenvironmental cues for DC development than those available to other peripheral tissue-resident DC precursors (reviewed in [55]) [56]. Fortunately, the ongoing search for thymic seeding progenitors has resulted in the progressive elucidation of putative tDCs precursors as well. The populations that are thought to seed the thymus include multipotent progenitors (MPPs), lymphoid-primed multipotent progenitors (LMPPs), CLPs, and circulating T cell progenitors (CTPs) [57]. Early studies showed that the majority of thymic and splenic pDCs had undergone IgH gene D-J rearrangements, and that they expressed CD3 and preT*α*, which provided evidence for DC development from CLPs or a similar precursor [58]. A minority population of CD8^+^ tDCs also exhibited these characteristics, which would coincide with the low percentage of CD8^+^ tDCs labeled in the IL-7R fate-mapping experiments [59]. Overall, it appears that cDCs do not arise from a CLP or CLP-similar precursor, whereas pDCs likely do. 

### 2.4. Intrathymic Precursors of tDCs

The ability of some T cell precursors to develop into DCs when removed from the thymus has suggested that these cells could be physiological precursors of tDCs. T cell precursors within the thymus are characterized as double negative (DN; CD8^−^ CD4^−^) and develop from DN1 (CD44^+^ CD25^−^) into DN2a cells (c-Kit^hi^ DN44^+^ CD25^+^), which is the point of T cell specification. DN2a cells retain the ability to differentiate *in vitro* into natural killer (NK) cells and DCs [60, 61]. Next, DN2a cells give rise to T-lineage committed DN2b cells (c-Kit^+^ CD44^+^ CD25^+^) and eventually differentiate to DN3 cells (c-Kit^−^ CD44^−^ CD25^+^), which must receive survival signals through the pre-T cell receptor to progress further through T cell development. The DN1 cells can be further subdivided into early T cell progenitors (ETPs; DN1a/b; c-Kit^hi^ CD24^−/lo^), DN1c (cKit^int^ CD24^hi^), DN1d (cKit^−^ CD24^+^), and DN1e (cKit^−^ CD24^−^) subsets based on their surface expression of c-Kit and CD24 [62]. ETPs are the canonical T cell precursors and contain some NK cell potential, whereas DN1c and DN1d cells exhibit B cell potential. Little is known about the lineage potential of DN1e cells. 

Many studies have provided evidence that T cell precursors have DC [63] and myeloid [64, 65] lineage potential. During specification, T-lineage genes are upregulated, and genes influencing development towards other lineages are downregulated. Interestingly, the minimal myeloid potential present in DN1 subsets is lost in DN2 cells, whereas DC potential is still present in DN2 cells which have not yet upregulated the T cell specific gene, *lck* [63]. Moreover, numerous *in vivo* studies have shown that intrathymic precursors, prior to T cell commitment at the *β*-selection checkpoint, can develop into tDCs [35, 49, 66]. 

Additional *in vivo* studies have supported the ability of distinct T cell precursors to give rise to DCs. Early studies characterized a “low-CD4 precursor” (CD4^lo^ CD8^−^ CD3^−^ CD24^hi^), which contained what are now referred to as DN1c and DN1d cells, that could give rise to CD8^+^ tDCs following intravenous injections into irradiated mice [49]. One progenitor within the thymus expressing CD24, c-Kit, CD11c, and Langerin can arise from MDPs, CDPs, and pre-DCs from the bone marrow and spleen and has been shown to give rise to Langerin^+^ CD8^+^ tDCs [25]. Studies by our laboratory have shown that ETP, DN1d, and DN1e subsets can all give rise to tDCs *in vivo*, which localize to the medulla in nonirradiated mice [35]. Unquestionably, there are many developmental routes by which DCs can arise, depending on a variety of factors such as their localization and surrounding stimuli, which in turn influences the transcriptional regulators that orchestrate cellular fate.

## 3. Context-Dependent Transcriptional Regulators of Lymphoid Tissue-Resident DCs

Despite the differences in the location of DC development, specific subsets share transcriptional regulatory programs, which indicates an intrinsic requirement for certain transcription factors for the DC lineage [67]. Interestingly, to date there is no known single transcription factor that is universally required for the development of all DCs, analogous to the requirement of Pax-5 for the development of all B cells [68], highlighting the versatility and plasticity of DC development and homeostasis. 

### 3.1. The Multitasking Transcriptional Regulators: Ets Transcription Factors

#### 3.1.1. PU.1

The two Ets transcription factor family members PU.1 and Spi-B have been intensely studied in myeloid and lymphoid cells owing to their expression in many progenitors and their roles in multiple lineages. PU.1 is expressed during the earliest stages of hematopoiesis onwards in CMP, CLP, CDP, preDC, DN1 cells, cDCs, and pDCs [37, 69, 70]. Early studies of the functions of PU.1 in DCs were conflicting due to the generation of two independent lines of PU.1 knockout mice, one of which was embryonic lethal, whereas the other one allowed survival until about two weeks after birth [71, 72]. Neither PU.1-deficient mouse strain, however, enabled analysis of the adult splenic and thymic DC compartments which are established 3–5 weeks after birth [73]. PU.1 (*Spi-1*)-deficient E14.5 and E16.5 embryos exhibited a lack of CD11c^+^ CD8^−^ tDCs, while CD11c^+^ CD8^+^ tDCs remained intact in one study [71]. However, another study demonstrated a reduction in DEC-205^+^ tDCs (equivalent to CD8^+^ tDCs; see Table 1) in 10- to 12-day old mice [72]. Subsequently, a polyI:C inducible PU.1-knockout clarified the requirement for PU.1 in splenic and thymic cDC and pDC populations and in the generation of these subsets from CDPs [74]. However, the involvement of PU.1 in other DC subsets and the generation of upstream precursors remain unclear. Interestingly, the context-dependent roles of PU.1 are emphasized by its ability to upregulate Flt3 in DCs [74], while exhibiting an equally important role in upregulating IL-7R in B cells [75]. Moreover, the dose of PU.1 is critical for lineage determination, as highlighted by a higher level of PU.1 favouring macrophage development over B cell and granulocyte development [76, 77]. PU.1 also plays a role in the macrophage/DC lineage decision, in part by binding to and inhibiting Mafb, which is a bZip transcription factor that promotes macrophage and monocyte development [78].

The roles of PU.1 in early thymocyte development are complex. PU.1 inhibits T cell development from DN2 cells [79] but is required for the generation of T cell precursors [80]. Interestingly, there is an accumulation of CD24^hi^ cKit^int^ Sca1^−^ DN1 precursors, corresponding phenotypically to the DN1c population, in PU.1^−/−^ animals [80], suggesting that it is needed for the developmental progression of DN1c cells to CD8^+^ tDCs. PU.1 induces the expression of many DC-promoting factors, such as M-CSFR, GM-CSFR, and CD11b [26, 27, 81, 82]. Thus, the decrease of PU.1 during early T cell development correlates with the loss of DC potential and likely results in the downregulation of a DC-specific gene program. The complexity of the functions of PU.1 in the intrathymic T/DC lineage choice is highlighted by a recent study, which amalgamated global transcript analysis with chromatin structure data over the early stages of T cell development. These results revealed, surprisingly, that during the stages of PU.1 expression from DN1 to DN2b cells, there were just as many targets of PU.1 in T cells as there were in B cells and macrophages. Importantly, however, these targets were unique and corresponded to genes active in early T cell development [83]. Therefore, PU.1 plays very important but divergent roles in DC and T cell development, by coordinating the expression of target genes required for each lineage. The ability of PU.1 to direct T-lineage gene expression is likely due to collaboration with Notch signals [84]. Other factors that may collaborate with PU.1 in the T/DC choice are under investigation.

#### 3.1.2. Spi-B

Spi-B is another Ets family transcription factor that is closely related to PU.1. Initially, Spi-B was identified as a lymphoid-specific factor involved in B cell receptor signaling [85]. Surprisingly, however, a knock-in of Spi-B into the PU.1 locus showed that it was able to rescue myeloid but not B cell development [86], and it was subsequently found to be expressed specifically in pDCs [87]. Further studies using RNA interference techniques showed that Spi-B is required for pDC development from human precursors [88], and it has recently been shown to be influential in bone marrow-derived pDC development [89]. Curiously, Spi-B does not appear to play a role in the generation of splenic pDCs, suggesting that its main roles are developmentally upstream of the immature DC precursors found in the spleen. Interestingly, Spi-B activates the production of type I IFN in concert with interferon regulatory factor-7 (IRF-7), a factor important for pDC function [89]. Unlike PU.1, which is normally expressed in DN1 and DN2 cells and decreases as T cells develop, Spi-B increases in expression during the DN1-3 stages, suggesting a role in T cell commitment [70]. Furthermore, Spi-B^−/−^ animals exhibit slightly lower cellularity and delayed T cell development in the thymus. However, overexpression of Spi-B at the DN3 stage interrupts *β*-selection resulting in greater DC development within fetal thymic organ culture (FTOC) [90] and inhibits T cell, B cell, and NK cell development from human precursors *in vitro* [87]. The impact of Spi-B overexpression on lymphocyte development may be due to the levels driven by PU.1-locus regulatory elements or retroviral elements in these studies, enabling Spi-B, which binds to the same promoter site as PU.1, to act in a PU.1-like manner. The presence of DC subsets therefore in PU.1^−/−^ and Spi-B^−/−^ mice is further evidence of a compensatory role for these two factors. Accordingly, there is a complete lack of tDCs in PU.1^−/−^ Spi-B^−/−^ E18 fetal thymic lobes in contrast to a reduction of DC subsets in PU.1^−/−^ lobes [90]. Adult Spi-B^−/−^ tDCs, however, appear normal (unpublished data), suggesting that PU.1 is capable of compensating for a loss of Spi-B specifically in tDCs, whereas the reverse relationship is not present.

### 3.2. Controlling the DC versus Macrophage Lineage Choice

#### 3.2.1. Ikaros

Ikaros is a zinc finger transcription factor that acts as a dimer with itself and with the other family members, Aiolos and Helios. Ikaros is critical for early stages of hematopoiesis [91], which has complicated analysis of developmental defects in different lineages in Ikaros-deficient mice. Ikaros dominant negative mutant mice, which lack activity of all Ikaros family members, exhibit a loss of cDCs and an increase in monocytes and macrophages [92], suggesting a requirement for Ikaros in cDC development. Interestingly, however, Ikaros null mice only lack CD8^−^ cDCs and pDCs, while retaining their CD8^+^ DC population, indicating that Ikaros is either needed in each lineage independently or that Ikaros null CD8^+^ DCs arise independently of the CDP. In another mouse model in which only low levels of Ikaros were expressed in hematopoietic cells only, pDCs were absent, indicating that pDCs require high levels of Ikaros whereas cDCs do not [93]. This defect was cell autonomous and was linked to inappropriate upregulation of a large array of genes and a failure to respond to Flt3L. Interestingly, Flt3 expression was missing in Ikaros null LMPP cells [94]. Therefore, part of the role of Ikaros in pDCs is to silence alternative lineage genes and to upregulate Flt3 on DC precursor populations. Interestingly, Ikaros can bind to promoter elements in the PU.1 gene locus to activate or repress PU.1 transcription in myeloid cells, depending on the regulatory site [95]. Overall, these data support a role for Ikaros in pDC development as well as the divergence of the cDC and monocyte-derived DC lineages prior to the CDP stage of DC development.

#### 3.2.2. Gfi1

Gfi1 is another transcriptional regulator with important roles in DC development. One of the main roles of Ikaros in the B/macrophage lineage choice is to upregulate Gfi1, promoting B cell development and repressing myeloid development [32]. It is therefore possible that Gfi1 is downstream of Ikaros in DCs as well. However, Gfi1^−/−^ mice exhibit a more striking DC deficiency than Ikaros^−/−^ mice, with a reduction in all splenic, thymic, and peripheral LN DC populations, correlated with an increase in LCs [96]. Gfi1^−/−^ mice also exhibit defects in early T cell development, reduced thymic cellularity, and increased Id2 mRNA levels [33]. Gfi1 represses Id2 in B and myeloid cells. This might also occur in developing T cells, since it is expressed throughout T cell development [97, 98]. In the context of multipotent progenitors, Gfi1 promotes the B cell lineage over the macrophage lineage by repressing PU.1 [32]. Moreover, *in vitro* experiments showed an increase in macrophage potential from Gfi1^−/−^ precursors. Collectively, these results indicate that Gfi1, like Ikaros, likely play a role in the DC/macrophage lineage choice.

### 3.3. cDC-Specific Regulators

#### 3.3.1. Zbtb46

Recently, two independent studies identified a novel transcription factor, Zbtb46 (also known as Btbd4 or zDC), exclusively expressed in pre-cDC, CD8^+^ cDC, and CD8^−^ cDC cells, but not in pDCs [99, 100]. Although Zbtb46 expression was restricted to these lineages, it was not required for their development, but rather to modulate their activation status [100–102]. Zbtb46 acts primarily as a transcriptional repressor in cDCs, with targets including many MHC class II genes. Once cDCs are stimulated with TLR agonists, Zbtb46 protein is downregulated, allowing MHC class II molecules to be expressed at higher levels, thereby conferring an activated status to these cDCs [102]. Zbtb46 might also play a role in promoting the development of CD8^+^ cDCs over CD8^−^ cDCs in the spleen [102]. However, the deletion of Zbtb46^+^ cells using diptheria toxin did not affect tumour or parasitic immunity, thus illuminating the compensatory roles of the remaining DC compartment in these functional capacities [100]. Certainly, the ability to label Zbtb46-expressing cells with GFP has provided a valuable tool for clarifying DC classification and enabling the identification of cells committed to the cDC lineage fate.

#### 3.3.2. Bcl6

Bcl6, another zinc finger transcription factor, is also known to be a transcriptional repressor [103, 104] of many target genes, including p53 [105]. This transcriptional regulator is involved in modulating Th2 immune responses [106, 107] and inhibiting plasma cell differentiation [108] and has recently been implicated in DC development [109]. Bcl6^−/−^ mice exhibit a reduction in the splenic CD4^+^CD8^−^ and CD8^+^ subsets. Additionally, as shown by adoptive transfer studies, Bcl6^−/−^ BM-derived precursors possessed a decreased capacity to develop into cDCs. This was attributed to increased p53 expression, leading to increased apoptosis [109]. Bcl6^−/−^ DCs also secreted greater amounts of IL-6 and IL-12, which led to a greater activation of CD4^+^ T cells, likely skewing to a Th2 inflammatory response [109]. Thus, Bcl6 plays a role in the differentiation and survival of cDCs.

### 3.4. Controlling the cDC versus pDC Lineage Choice

#### 3.4.1. Id2

Id factors, which contain helix-loop-helix domains, can dimerize with and inhibit E proteins including HEB (HEBAlt, HEBCan), E2A (E12, E47), and E2-2 (E2-2Can, E2-2Alt). The major cDC-specific Id regulator is Id2. Id2 is not expressed in LSK, LMPPs, or CLPs, or in the CDP or pre-cDC DC progenitors, but is present in all cDCs, regardless of anatomical location [110]. However, Id2 is only required for epidermal LCs, splenic CD8^+^, and nonlymphoid tissue resident CD103^+^ DCs [48, 111]. Interestingly, the DN1e subset within the thymus also expresses high levels of Id2 indicating that these cells might have an increased propensity to develop into cDCs, in particular CD8^+^ tDCs [35]. Thus, Id2 appears to have a role in the later stages of DC development. However, unlike Zbtb46, Id2 expression is not restricted to the DC lineage, since it is also important for the development of other lineages, such as NK and myeloid cells.

#### 3.4.2. E Proteins

In contrast to cDCs, pDCs require the E protein E2-2 for their development and homeostasis [34]. Interestingly, E2-2 can activate pDC-specific regulators, such as Spi-B, IRF-7, and IRF-8, as well as Bcl11a. Furthermore, the deletion of E2-2 from pDCs converts them to cDCs, as determined by surface marker phenotype, function, gene expression, and morphology [34, 112]. Since E2-2-dependent upregulation of these genes would be inhibited by Id2, the Id2/E2-2 dichotomy is likely at the top of the hierarchy that splits the pDC/cDC gene programs. Another E protein that is expressed specifically in thymic pDCs is HEBCan [35]. HEBCan is also expressed throughout thymocyte development, while the shorter form of HEB, HEBAlt, is expressed only during early T-lineage developmental stages. HEBAlt has defined roles in promoting T cell development [113, 114], and decreasing DC development from bone marrow precursors *in vitro* [35]. However, constitutive expression of HEBAlt in T cell precursors does not alter tDC development in the adult thymus, perhaps due to additional microenvironmental factors present in the thymus that are not available *in vitro* (A. J. Moore and M. K. Anderson, unpublished data). Therefore, further study is needed to assess the roles of HEBCan and HEBAlt in the T cell/tDC lineage choice.

### 3.5. CD8^+^ DC-Specific Regulators

#### 3.5.1. Batf3

Global gene expression analyses of DC populations have led to the discovery of many DC subset-specific genes, including the transcription factor Batf3 [7]. Studies of Batf3-deficient mice showed that Batf3 is required for CD8^+^ cDC development during steady state. The lack of splenic and LN CD8^+^ cDCs in Batf3^−/−^ mice demonstrated that these cells are required for cross-presentation of antigen to CD8^+^ T cells. Furthermore, these mice had defective antiviral and antitumor immunity [7]. Interestingly, Batf3 was also required for the generation of CD103^+^ CD11b^−^ DCs within the skin and mesenteric LN, dermis, lung, and intestine, which emphasizes the similarities in transcriptional regulation between CD8^+^ cDC and CD103^+^ nonlymphoid tissue DCs [115]. *In vitro* studies showed that the cultured equivalents to CD8^+^ DCs were not hampered by a lack of Batf3 until later timepoints, suggesting more of a homeostatic role than a developmental role of Batf3 in CD8^+^ DC development and also foreshadowing recent work highlighting the redundancy of Batf factors [110]. Interestingly, when challenged by intracellular pathogens or administration of IL-12, CD8^+^ DCs were restored by 3 weeks in Batf3^−/−^ mice by an alternative pathway whereby Batf and Batf2 compensate for the lack of Batf3 [31]. This study also showed that Batf could interact directly with IRF-4 and IRF-8. Thus, it appears that Batf3 is important in the terminal stages of CD8^+^ cDC development and plays a role in maintaining this subset.

#### 3.5.2. E4BP4

Recently, E4BP4 (NFIL3), a basic leucine zipper transcription factor, which was first recognized for its importance in NK cell development [116, 117], has been implicated in CD8^+^ DC development. Despite higher E4BP4 mRNA expression levels in pDCs than CD8^+^ cDCs, E4BP4^−/−^mice specifically lacked splenic and thymic CD8^+^ cDCs [30]. The defect in development appears to take place at the pre-cDC to CD8^+^ cDC developmental transition since precursors, such as LSK, CLP, CMP, GMP, CDP, and pre-cDC populations, are not affected by the absence of E4BP4 [30]. *In vitro* studies showed that E4BP4^−/−^ bone marrow cells could be partially rescued by retroviral transduction with a Batf3-containing vector into CD24^+^ Sirp*α* ^−^ DCs (CD8^+^ cDC equivalent), thus indicating that Batf3 is involved directly or indirectly with the CD8^+^ DC-promoting effects of E4BP4 expression.

### 3.6. CD8^−^ DC-Specific Regulator: RelB

Despite the identification of many regulators for the CD8^+^ cDC and pDC lineages, the regulation of the CD8^−^ cDC subset by unique transcription factors remains elusive. Initially, tDCs were reported absent in RelB^−/−^ mice, but this was attributed to a lack of medullary thymic epithelial cells which tDCs normally localize to [118, 119]. RelB, a subunit of the NFkB complex, is a downstream signaling mediator of immune cell activation via pattern recognition receptors, such as Toll-like receptors [120]. RelB is specifically expressed in splenic CD8^−^ cDCs and is required for their development [119]. Although functional roles pertaining to DC activation have been attributed to RelB in DCs [121, 122], the influence RelB has on lineage decisions is largely unknown. 

### 3.7. Interferon Regulatory Factors

As their names suggest, IRFs are transcription factors known for their ability to induce the expression of interferons in response to stimulus, such as the activation of toll-like receptors (reviewed in [123]). IRF-1, IRF-2, IRF-4, IRF-7, and IRF-8 have been implicated in DC development across many subsets.

#### 3.7.1. IRF-8

In addition to Batf3, Id2, and E4BP4, CD8^+^ cDCs also require IRF-8 (ICSBP; interferon consensus-binding protein) for their development [124, 125]. IRF-8 also plays a major role in CD103^+^ DCs and a minor role in pDC, LC, and dermal DC development with a more pronounced defect in pDCs [48, 124]. IRF8^−/−^ mice were unable to produce type I IFNs following viral challenge and exhibited delayed migration of LCs to the draining LNs in steady state and inflammatory conditions [124, 126, 127]. Interestingly, a single point mutation within the IRF association domain (IAD) of IRF-8, which confers the ability to interact with other IRFs, replicates the loss of CD8^+^ cDCs, but not pDCs, in IRF-8^−/−^ mice. Although the wildtype IRF-8 could interact with IRF-2 or PU.1 and Spi-B to bind to interferon-stimulated response element (ISRE) or Ets/IRF promoter sites, respectively, the mutated IRF8^R294C^ could not [128]. Therefore, IRF-8 is involved in the development of CD8^+^ cDCs, CD103^+^ DCs, and pDCs but likely act through different mechanisms in each subset. 

#### 3.7.2. Other IRFs

Another factor implicated in DC development is IRF-4. IRF-4-deficient mice lacked the majority of splenic CD11b^+^ CD4^+^ CD8^−^ cDCs and had a slight reduction in pDCs [129, 130]. In addition to developmental defects, the lack of IRF-4 impaired the migration of LCs and CD103^+^ dermal DCs to the cutaneous LN following skin inflammation [131]. IRF-1^−/−^ mice also differ from wildtype mice in that they exhibit a slight reduction in CD8^+^ and CD8^−^ cDCs and an increase in pDCs [132]. Further complexity is added by the severe decrease of CD8^−^ cDCs and a partial lack of CD8^+^ cDCs and pDCs in IRF-2^−/−^ mice [133]. Interestingly, IRF-4 mRNA expression levels were greater in E4BP4^−/−^ pre-cDCs compared to the wildtype counterparts, suggesting that E4BP4 might act by restricting the IRF4-mediated development of other DC lineages [30]. Thus, in addition to IRF-8, IRF-1 and IRF-2 play minor roles in CD8^+^ DC development, whereas IRF-2, IRF-4, and, to a lesser extent, IRF-1 are important for CD8^−^ DC development. The increase in pDCs in IRF1^−/−^ mice suggests that IRF-1 might repress or inhibit IRF-8. IRF-2 and IRF-4 also play minor roles in pDC development. Interestingly, ChIP analysis has shown that human E2-2, which is required for pDC development, is capable of binding to promoter regions upstream *Irf*-7 and *Irf*-8 gene loci [34].

## 4. Cytokines Involved in DC Development

### 4.1. GM-CSF, M-CSF, and Flt3

Cytokines, secreted by surrounding tissues and immune cells, provide many developmental cues that influence the transcriptional regulation and functions of the receiving cells. Initial *in vitro* studies of cytokines in DC development revealed distinct and important roles for the receptor tyrosine kinases, GM-CSF, M-CSF and Flt3L, in the generation of DCs [134–138]. Flt3L and M-CSF, in particular, have been shown to influence many discrete DC subsets. Flt3L-supplemented cultures can induce the differentiation of CD8^+^cDCs, CD8^−^ cDCs, and pDCs from a variety of precursors [23, 135–137, 139]. M-CSF-supplemented cultures can also generate CD8^+^ cDCs, CD8^−^ cDCs, and pDCs, albeit with lower efficiency than Flt3L cultures [138]. Moreover, Flt3^+^ precursors including LMPPs, MDPs, CDPs, pre-cDCs and a proportion of CLPs, CMPs, and ETPs, in addition to progenitors transduced to express Flt3, possess greater DC potential than their Flt3^−^ counterparts [42, 139–142]. Correspondingly, Flt3-deficient mice exhibit decreased cDCs and pDCs [41]. However, the degree of reduction in cDC and pDC subsets in Flt3^−/−^ mice does not reflect the severe decrease of these populations in Flt3L^−/−^ mice [23, 143], suggesting the presence of another, as of yet unidentified, receptor for Flt3L.

Interestingly, this speculation reflects recent findings in the M-CSF/M-CSF1R pathway. Mice carrying a mutated M-CSF gene (*op/op* mice) exhibited a reduction in splenic CD11c^dim^ B220^+^ pDCs, but LCs and microglia remained intact [144–146]. Microglia, the resident macrophages within the central nervous system (reviewed in [147]), and some LCs arise from progenitors in the embryonic yolk sac and thus exhibit similar developmental requirements [146, 148]. By contrast, LCs and microglia were completely absent from M-CSF1R^−/−^ mice [146, 149]. The disparity in DC developmental defects in M-CSF^−/−^ and M-CSF1R^−/−^ mice was clarified by the discovery of an alternate ligand for M-CSF1R, IL-34 [150]. IL-34 is secreted by keratinocytes and neurons to foster the development of steady state LCs and microglia, respectively [151]. Accordingly, IL-34^−/−^ mice lack LCs and exhibit reduced microglia, thereby replicating the results in M-CSF1R^−/−^ mice [151]. Comparable populations of monocytes and DCs were observed between IL-34^−/−^and WT mice [152]. By contrast, there are no significant LC deficiencies in Flt3^−/−^ or Flt3L^−/−^ mice [48, 153]. In addition to M-CSF1R expression on MDPs and CDPs, it is also expressed by yolk sac macrophages, adult macrophages, LCs, and splenic cDC and pDC subsets [145, 154]. Although Flt3 and M-CSFR are both expressed on MDPs and CDPs, they clearly influence different DC lineage fates.

Although GM-CSF is commonly added to many *in vitro *cultures to stimulate DC development from bone marrow progenitors, GM-CSF^−/−^ and GM-CSFR^−/−^ mice do not show any significant deficiencies in DC populations in lymphoid tissues [155]. Splenic CD8^+^ cDCs were slightly increased in GM-CSF^−/−^ mice, indicating that GM-CSF inhibits the generation of this subset [156]. There are many conflicting reports on the involvement of GM-CSF in nonlymphoid tissue DC subsets. One study shows that CD103^+^ CD11b^−^ dermal DCs are reduced in GM-CSF^−/−^ mice and GM-CSFR^−/−^ mice [157], which is confirmed by another report, whereby CD103^+^ CD11b^+^ lamina propria DCs and CD103^+^ DCs from skin and lung draining LN were also decreased in both GM-CSF^−/−^ and GM-CSFR^−/−^ mice [158]. A third report observed that DC populations remained similar to WT in GM-CSFR^−/−^ mice, but CD103 surface expression was slightly downregulated on GM-CSFR^−/−^ DCs [159]. Although GM-CSF does not seem to be unequivocally required for many, if any, DC subsets, GM-CSFR transgenic mice exhibit an increase in cellularity in the thymus and spleen, which is echoed by an increase in cDCs as well [155, 156]. Conversely, the presence of GM-CSF inhibits the development of CD8^+^ cDC equivalent cells and pDCs *in vitro* [136, 156]. Moreover, GM-CSF does not enhance DC development from early T cell precursors as Flt3L does [160]. GM-CSF does, however, seem to play a role in the function of DCs. The addition of GM-CSF to *in vitro* cultures resulted in the upregulation of CD103 and an increase in cross-presentation abilities of DCs [161], which was confirmed *ex vivo *and *in vivo* using GM-CSF-transgenic and GM-CSFR^−/−^ mice [162]. 

 Therefore, GM-CSF signaling directs different developmental outcomes than Flt3L signaling. Although many other cytokines, such as SCF, TGF-*β*, IL-3, IL-4, or IL-7, have been studied and can modify the outcomes of *in vitro* cultures, they do not appear to play an overarching, essential role for DC development.

### 4.2. STATs

The signal transducer and activator of transcription (STAT) family of transcription factors has been implicated downstream of the cytokine receptors, Flt3 and GM-CSFR, thus bridging the gap between extracellular signals and transcriptional regulation. Signaling through the Flt3 receptor induces the phosphorylation of STAT3, which is required for DC development as evidenced by the lack of splenic DCs and reduced CLP and CMP precursors in STAT3^−/−^ mice [28]. This defect was not restored by treating mice with Flt3L, indicating that the requirement for STAT3 is downstream of Flt3 signaling [28]. STAT1, STAT3, and STAT5 are all phosphorylated in response to administration of GM-CSF to bone marrow cultures [28]. GM-CSF blocks pDC development *in vitro* through STAT5, which inhibits IRF-8 transcription [29]. Clearly, the Flt3L and GM-CSF pathways are connected, since Flt3 can induce the transcription of GM-CSFR, as well as M-CSFR and PU.1 [142]. Thus, this experimental evidence suggests that Flt3 is required during earlier stages of DC development, whereas the function of GM-CSF might be to favour the cDC lineage over pDCs. The point in DC differentiation at which M-CSF influences developmental outcomes is likely during the MDP to CDP conversion when M-CSF1R is expressed, but this has not yet been directly examined. Determining the cellular sources of Flt3L, GM-CSF, and M-CSF will provide important insights into the homeostatic versus infection-induced mechanisms of DC development. 

## 5. cDC and pDC Gene Regulatory Networks

**Figure 1:**
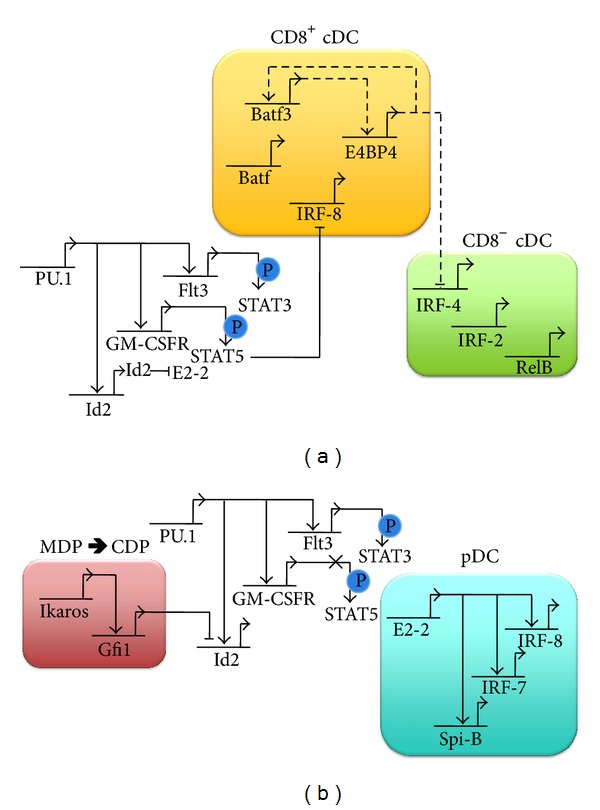
Gene regulatory networks for cDC and pDC development. Shared gene regulation patterns in (a) and (b). PU.1 upregulates many factors important for DC development, including Id2, GM-CSFR, and Flt3L [26, 27]. The Flt3 pathway phosphorylates STAT3, which can upregulate/downregulate target genes [28]. (a) Gene regulation in cDCs. Id2 expression inhibits E2-2 via protein interaction. GM-CSFR phosphorylates STAT5, which can inhibit IRF-8 expression [29]. Batf3 upregulates E4BP4 [30]. Batf expression in CD8+ cDCs compensates for a lack of Batf3 [31]. E4BP4 negatively modulates IRF-4 expression [30]. (b) Gene regulation in pDCs. Ikaros upregulates Gfi1 [32], which can inhibit Id2 expression [33], allowing for E2-2 function. E2-2 binds to the promoter of Spi-B, IRF-7, and IRF-8 to upregulate gene expression [34]. A yet unidentified mechanism prevents the downstream events of GM-CSFR in pDCs, since STAT5 has been shown to downregulate IRF-8, which is required for pDC development. Proven interactions are indicated in solid bars. Hypothesized interactions are shown in dashed lines.

Once organized into lineage-specific gene regulatory maps, the similarities and differences between cDCs and pDCs become more apparent (Figure 1). The networks are separated based on the stage of development in which each factor is proposed to function. PU.1 is a master regulator of both cDCs and pDCs, and, based on experimental evidence, it likely functions early in DC development at or immediately prior to the CDP stage. The main function of PU.1 is to turn on regulatory genes that are responsible for proper DC development, such as Id2, Flt3L, and GM-CSFR. Since signaling through GM-CSFR can activate STAT5, which inhibits IRF-8 transcription, GM-CSF might be an environmental cue to favour CD8^−^ cDC development. Indeed, GM-CSF promotes the development of CD8^−^ CD11b^+^ DCs *in vitro* [29]. The partial restoration of a wildtype phenotype by transducing E4BP4^−/−^cells with Batf3 suggests that either E4BP4 and Batf3 have similar transcriptional targets or Batf3 is upregulated by E4BP4. Conversely, the elevated levels of IRF-4 mRNA in E4BP4^−/−^ cells indicate that E4BP4 inhibits IRF-4, directly or indirectly (Figure 1(a)).

Clearly, Id2 functions to inhibit pDC development by binding to and inhibiting E2-2, which is required for pDCs. Although the earlier Ikaros mutant studies were contradictory, a model in which Ikaros is expressed only at low levels elucidates its role in the pDC lineage. In this model, Ikaros upregulates Gfi1, and Gfi1 inhibits Id2 transcription (Figure 1(b)). The repression of Id2 would result in functioning E2-2 protein, which can reprogram precursors for the pDC lineage fate by upregulating Spi-B, IRF-7, and IRF-8. There must be mechanisms in place to restrict GM-CSF signals from inhibiting IRF-8 through STAT5 to allow for CD8^+^ cDC development, as well as pDCs. Future studies examining the environmental cues and resulting transcriptional regulation will allow us to further understand the mechanisms that govern homeostatic DC development and infection- or inflammatory-induced DC differentiation.

**Figure 2:**
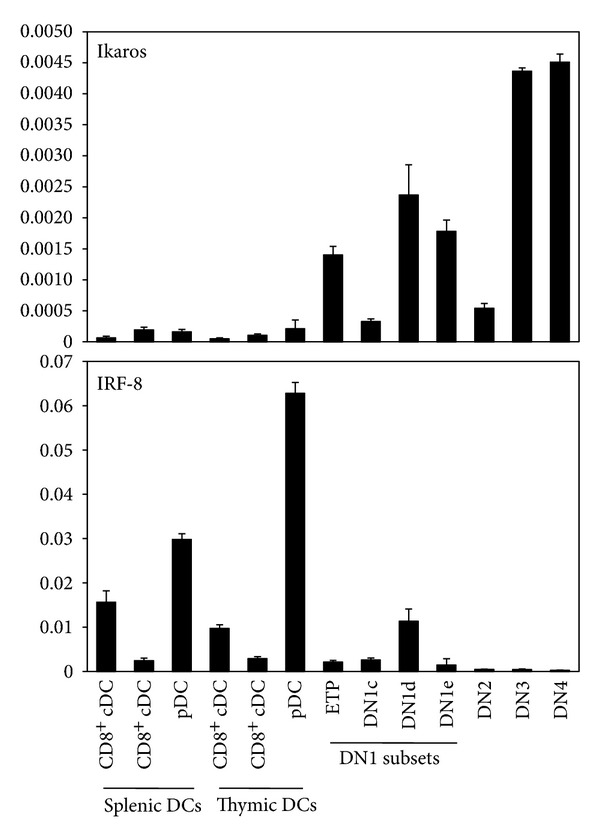
IRF-8 and Ikaros gene expression in early T cell precursors. Cell subsets were sorted, and qRT-PCR was performed as previously described [35]. Gene expression levels, as determined by qRT-PCR, were normalized to *β*-actin. Values shown are mean ± standard deviation (*n* = 3).

Many of the major DC regulators, such as PU.1, Spi-B, Gfi1, Id2, and IRF-4, are expressed by developing T cell precursors [70, 163]. However, with the exception of PU.1, the gene targets and roles of each factor have not been explored in T cell progenitors versus DC progenitors. Here, we examined the gene expression profiles of Ikaros, IRF-8, and Batf3 in ETP, DN1c, DN1d, DN1e, DN2, DN3, and DN4 cells to determine whether DC gene network components were present in these precursors (Figure 2). Batf3 was not expressed at high levels, if at all, in these T cell precursors (unpublished data). However, Ikaros was expressed and increased as precursors became committed to the T cell lineage (Figure 2). Earlier work showed that fetal T cells, but not adult T cells, were absent from Ikaros null mutant mice [164]. The presence of Ikaros could upregulate Gfi1, which is known to be expressed in T cell precursors, to inhibit Id2 and promote pDC development. Interestingly, mature splenic and thymic DC subsets do not express high levels of Ikaros or Gfi1 (Figure 2; unpublished data), agreeing with the speculation that Ikaros and Gfi1 play roles early in DC development but not in mature DCs. DN1d cells, which we have previously determined, express high levels of Spi-B [35], contained the highest levels of IRF-8 when compared to the remaining T cell precursors (Figure 2). These results indicate that DN1d cells might have a greater pDC lineage potential. Overall, the expression of multiple DC-essential transcription factors within T cell precursors suggests these cells are partially equipped to develop into DCs.

## 6. Discussion

Although the properties varying between distinct DC subsets are vast, there is emerging evidence linking DC populations by common gene expression profiles [67]. These comparisons show that lymphoid tissue-resident CD8^+^ cDC and nonlymphoid tissue-resident CD103^+^ DCs are more closely related to each other than they are to CD8^−^ cDCs and pDCs. Similarly, migratory DCs differ from all other DC subsets and uniquely upregulate genes expressing immunomodulatory molecules, which could regulate immune response to self-antigen [67]. It is probable that the transcriptional regulators expressed earlier in DC development, such as PU.1, Ikaros, and Gfi1, primarily function to modulate precursor responsiveness to cytokine signals, growth factors, and inflammatory signals. These events allow for the production of steady state DC subsets and prompt alternative pathways of DC development during infection [31, 165]. By contrast, while the transcription factors expressed during the terminal stages of DC differentiation might be required for DC subset development, they are often also essential for specialized functions. In particular, RelB^−/−^ and IRF-8^−/−^ DCs express lower levels of MHC class II and costimulatory molecules, such as CD40, CD80, and CD86, following microbial or CD40L stimulation [122, 125]. The tolerogenic cytokines TGF-*β* and IL-10 were secreted at higher concentrations from IRF-1^−/−^ DCs [132]. Furthermore, the transcriptional marker of cDCs, Zbtb46, has been shown to play important functions by promoting tolerogenic phenotypes of steady state cDCs until stimulated by antigen [102]. Certainly, the duality of these transcription factors for developmental and functional inputs makes targeted experiments more challenging to design. Despite the availability of many high throughput methods, such as RNA-seq or ChIP-seq, flaws in data interpretation can still arise from purifying DCs according to their surface cellular phenotypes. If a method for typing single cells by transcriptome signatures was available, it would be interesting to see how DC subsets that emerged from this analysis would compare with established DC subsets grouped by combinatorial cell surface receptor expression.

## Figures and Tables

**Table 1:** Surface molecule expression of steady state dendritic cell subsets. Phenotype of lymphoid-resident CD8^+^ cDC, CD8^−^ cDC, pDC, nonlymphoid tissue-resident CD11b^+^, CD103^+^, CD103^+^ CD11b^+^ DCs, and Langerhans cells. CD103^+^ CD11b^+^ DCs only exist in the lamina propria of the intestine. Transcription factors important for each DC lineage and known human DC equivalent subsets are listed. *Thymic CD8^+^ cDCs express Langerin. ^#^CD103^+^ DCs in the peyer's patches also express CD8*α*. Abbreviations, CD numeration, and alternate names: DEC-205 (CD205), B220 (CD45R), PDCA-1 (plasmacytoid DC Ag-1; CD317; Bst2), Siglec H (Sialic acid-binding immunoglobulin-like lectin H), Langerin (CD207), CD141 (BDCA-3), CD1c (BDCA-1), and CD303 (BDCA-2).

	CD11c	MHC class II	CD8*α*	CD11b	CD4	DEC-205	B220	PDCA-1	Siglec-H	CD103	Langerin	Master regulators	Minor regulators	Human DC subset equivalent
CD8^+^ cDC	+	+	+	−	−	+	−	−	−	−	−*	PU.1, Id2, Batf3, E4BP4, IRF-8, Flt3	Gfi1, IRF-1, IRF-2	CD11c^lo^ CD141^+^ CD11b^−^ XCR1^+^
CD8^−^ cDC	+	+	−	+	+/−	−	−	−	−	−	−	PU.1, RelB, Flt3	Gfi1, Id2, IRF-1, IRF-4, IRF-7	CD11c^hi^ CD11b^+^ CD1c^+^
pDC	int	int	−	−	−	+	+	+	+	−	−	E2-2, PU.1, Ikaros, IRF-8, Flt3	Spi-B, Gfi1, IRF-2	CD123^+^ CD303^+^ CD304^+^
CD103^+^	+	+	−^#^	−	−	+	−	−	−	+	+	Id2, Batf3, IRF-8		
CD11b^+^	+	+	−	+	−	+/−	−	−	−	−	−			
CD103^+^	+	+	−	+	−	+	−	−	−	+	−			
CD11b^+^														
Langerhans cells	int	+	−	+	−	+	−	−	−	−	+	Id2, M-CSFR	IRF-8	

CD1c = BDCA-1.

CD303 = BDCA-2.

CD141 = BDCA-3.

CD103^+^ are CD8^+^ in the peyer's patches.

CD103^+^ CD11b^+^ only in lamina propria.

## List of Abbreviations

DC: Dendritic cell
tDC: Thymic DC
cDC: Conventional DC
pDC: Plasmacytoid DC
LC: Langerhans cell
LNs: Lymph nodes
IPCs: Interferon-producing cells
CLP: Common lymphoid progenitor
CMP: Common myeloid progenitor
GMP: Granulocyte/macrophage precursor
MDP: Macrophage/DC precursor
CDP: Common DC precursor
MPP: Multipotent progenitor
LMPP: Lymphoid-primed multipotent progenitor
CTP: Circulating T cell progenitor
DN: Double negative
NK: Natural killer
ETP: Early T cell progenitor
FTOC: Fetal thymic organ culture
IFNs: Interferon regulatory factors
ICSBP: Interferon consensus-binding protein
IAD: IRF association domain
ISRE: Interferon-stimulated response element
Flt3L: Flt3 ligand
STAT: Signal transducer and activator of transcription.
